# Low-Cost Curb Detection and Localization System Using Multiple Ultrasonic Sensors

**DOI:** 10.3390/s19061389

**Published:** 2019-03-21

**Authors:** Joon Hyo Rhee, Jiwon Seo

**Affiliations:** 1 School of Integrated Technology, College of Engineering, Yonsei University, 85 Songdogwahak-ro, Yeonsu-gu, Incheon 21983, Korea; jnhyo@yonsei.ac.kr; 2 Yonsei Institute of Convergence Technology, Yonsei University, 85 Songdogwahak-ro, Yeonsu-gu, Incheon 21983, Korea

**Keywords:** autonomous vehicle, curb detection, curb localization, environmental recognition, ultrasonic sensor array

## Abstract

Curb detection and localization systems constitute an important aspect of environmental recognition systems of autonomous driving vehicles. This is because detecting curbs can provide information about the boundary of a road, which can be used as a safety system to prevent unexpected intrusions into pedestrian walkways. Moreover, curb detection and localization systems enable the autonomous vehicle to recognize the surrounding environment and the lane in which the vehicle is driving. Most existing curb detection and localization systems use multichannel light detection and ranging (lidar) as a primary sensor. However, although lidar demonstrates high performance, it is too expensive to be used for commercial vehicles. In this paper, we use ultrasonic sensors to implement a practical, low-cost curb detection and localization system. To compensate for the relatively lower performance of ultrasonic sensors as compared to other higher-cost sensors, we used multiple ultrasonic sensors and applied a series of novel processing algorithms that overcome the limitations of a single ultrasonic sensor and conventional algorithms. The proposed algorithms consisted of a ground reflection elimination filter, a measurement reliability calculation, and distance estimation algorithms corresponding to the reliability of the obtained measurements. The performance of the proposed processing algorithms was demonstrated by a field test under four representative curb scenarios. The availability of reliable distance estimates from the proposed methods with three ultrasonic sensors was significantly higher than that from the other methods, e.g., 92.08% vs. 66.34%, when the test vehicle passed a trapezoidal-shaped road shoulder. When four ultrasonic sensors were used, 96.04% availability and 13.50 cm accuracy (root mean square error) were achieved.

## 1. Introduction

Since Google obtained the first license for an autonomous vehicle from the state of Nevada in 2012 [1], several other companies have made remarkable progress in autonomous vehicle technology. For instance, in 2015, Audi announced that their prototype had successfully driven itself from Silicon Valley to Las Vegas [2]. It is also known that BMW is testing their autonomous driving technology in a prototype vehicle on highways between Munich and Nuremberg [3]. Furthermore, Volvo is cooperating with the government of Sweden in planning a project that will produce one hundred autonomous cars to drive on city streets [4]. Many other automobile companies and automotive component companies are rushing into the market of autonomous driving technology. For these reasons, the autonomous driving industry is currently drawing more attention than ever before.

Autonomous driving technology consists of various subcategories, one of the most important of which is environmental recognition, as this directly influences the safety of drivers and pedestrians. Techniques that use sensors to detect objects surrounding the vehicle fall under the heading of environmental recognition technology. Various driving situations correspond to a diverse list of potential surrounding objects to be recognized, such as adjacent vehicles, pedestrians, lanes and crossroads, traffic lights, signs, and curbs. 

The ability to detect and localize curbs is especially useful. By providing information regarding the outside boundaries of the driving lanes, curb detection and localization systems prevent vehicles from intruding into pedestrian walkways and can detect the lane in which the vehicle is driving. Driving lane detection is a crucial technology for autonomous driving. Usually, driving lane detection is primarily based on precise Global Navigation Satellite Systems (GNSS) positioning technology [5,6,7] and image processing from vision sensors [8,9,10,11,12,13]. GNSS, especially the Global Positioning System (GPS) of the U.S., are widely used for various transportation modes and applications [14,15,16,17,18,19]. However, the driving lane detection using GNSS requires a precise digital map. Otherwise, the absolute 3D position obtained by GNSS cannot be converted to the driving lane information. The primary weakness of GNSS stems from the system’s vulnerability to radio frequency interference [20,21,22,23,24,25,26,27,28] and ionospheric effects [29,30,31,32]. The performance of the vision sensor [33,34] can be impeded by environmental factors such as light and weather conditions [35,36,37]. Because of these factors, detecting a driving lane is not a simple task for autonomous vehicles. Thus, curb localization information could improve the performance of the current lane detection techniques. Curb localization information can also be used to implement an automatic pull-over system, which automatically pulls over the vehicle to the curb in an emergency situation such as a driver having a heart attack. To realize these benefits, several research groups have been developing various curb detection and localization systems.

Previous studies on curb detection and localization systems predominantly utilized light detection and ranging (lidar) sensors [38,39,40,41,42,43,44,45,46]. Lidar is a popular sensor that measures distance with a high accuracy by illuminating an object with a laser. Multichannel scanning lidar is used to obtain a 3D point cloud of the surrounding environment. In fact, lidar-based curb detection and localization systems usually utilize 32- or 64-channel lidar systems. The resolution of the 3D point cloud is improved by increasing the number of scanning channels. 

There have been many recent developments that attempt to improve the accuracy and resolution of curb detection and localization systems. For instance, Wang et al. [47] used a commercial mobile laser scanning system that consisted of two single-channel lidars, an inertial measurement unit, and GNSS equipment to detect road boundaries based on local normal saliency. Chen et al. [48] performed curb detection with a range of up to 50 m using a 64-channel lidar. Zhao et al. [49] adapted a particle filter to the measurements from a 64-channel lidar to detect curbs. Fernandez et al. [50] suggested a curb detection method using both 64-channel lidar and a stereo vision sensor. Lastly, Hata et al. [51] used a 32-channel lidar to recognize urban environments including curbs for vehicle localization.

Despite these benefits, multichannel lidar also has certain disadvantages such as high price and the existence of wide blind spots. Multichannel lidar is too expensive for practical use in commercial vehicles. The existence of wide blind spots is another weakness of scanning lidar. Most multichannel lidar systems are attached to the roof of the vehicle to facilitate a 360° view. However, because of the placement of the roof relative to the lidar system, the lidar cannot detect the region immediately surrounding the vehicle. This blind spot is not critical to detecting most nearby obstacles such as other vehicles or pedestrians, but it can become problematic in certain situations such as close approaches to curbs. For these reasons, the commercialization of curb detection and localization systems with a multichannel lidar constitutes a difficult task. Therefore, it is beneficial to consider using alternative sensors that are both cost-effective and able to reduce the extent of blind spots.

An ultrasonic sensor is a typical low-cost sensor that measures the distance to an object using ultrasonic sound waves. Such sensors contain an ultrasonic wave transmitter and receiver and calculate distance by multiplying the speed of sound and the duration of the wave propagation. Ultrasonic sensors are widely used in a variety of situations, ranging from daily tasks to industrial settings. For instance, vehicles equipped with parking assistance systems use ultrasonic sensors to determine whether there is enough space between adjacent vehicles. The widespread use of ultrasonic sensors can be attributed to their low price. More specifically, a typical ultrasonic sensor costs under $30, which is far less expensive than the lidar counterpart. 

Using ultrasonic sensors in curb detection and localization systems can increase the price competitiveness and thus offer a more practical commercial solution. However, the performance of ultrasonic sensors is significantly lower than that of the lidar counterpart. A single, vehicle-mounted ultrasonic sensor can only measure one-dimensional distance within a range of about 10 m. Furthermore, the data acquisition rate of an ultrasonic sensor is inevitably lower than that of a lidar because the speed of an ultrasonic wave is much lower than the speed of a laser. Given that the speed of sound in air at sea level is 340 m/s, the theoretical maximum data acquisition rate is 34 Hz when the target object is 5 m away from the ultrasonic sensor. The actual data acquisition rate of the ultrasonic sensor used in our testbed was 10 Hz. Because the data acquisition rate is low, the distance information becomes sparse so that even critical features of target objects might be missed. This problem decreases the detection performance of ultrasonic sensors. 

Therefore, novel approaches are required to improve the performance of ultrasonic-sensor-based curb detection and localization systems. To the authors’ knowledge, there is no literature reporting a curb detection and localization system based on ultrasonic sensors that has demonstrated satisfactory performance. This paper proposes a curb detection and localization system using multiple ultrasonic sensors with novel processing algorithms. This involves both hardware implementation of the testbed and the introduction of software algorithms. 

In Section 2, the implementation of our testbed is described. Section 3 presents several distance estimation algorithms for curb detection and localization. Because the performance of the distance estimation algorithms in Section 3 is unsatisfactory, Section 4 describes our series of processing algorithms to enhance the availability of reliable distance estimates while providing good distance-estimation accuracy. Results from a field test presented in Section 5 demonstrate the performance of the proposed algorithms, which was significantly better than the previous algorithms during four representative driving situations. Section 6 concludes this paper.

## 2. Testbed Implementation

The hardware of our curb detection and localization testbed consisted of four ultrasonic sensors, a single-channel lidar, a camera, a GPS receiver, and a laptop. The ultrasonic sensors measured the distance between the curb and the side of the vehicle. The single-channel lidar provided true distance measurements so that the accuracy of the proposed system can be evaluated. The camera recorded the view in front of the vehicle, including curbs. The GPS receiver collected the position and velocity data of the vehicle. However, the lidar, camera and GPS receiver were not a part of our curb detection and localization system. Instead, they merely verified the performance of the proposed system.

The ultrasonic sensor is a widely used sensor in various applications. In fact, ultrasonic sensors are already installed on most vehicles for parking assistance. In our testbed, three ultrasonic sensors, obtained at a cost of $15 each, were selected for implementing a low-cost curb detection and localization system. The sensor uses a 40 kHz ultrasonic wave to detect objects. 

An ultrasonic sensor can only provide a single measurement output for each transmitted ultrasonic pulse. Therefore, even if there are multiple objects in the detection range, a single ultrasonic sensor only perceives one object. This can create a problem, in that other unintended objects, e.g., the ground, can be noticed instead of the intended target, e.g., the curb. In practice, sudden data jumps in the distance measurements from ultrasonic sensors also cause a reliability problem. Measurement outliers exist for any distance measuring sensors, but, in driving situations, ultrasonic sensors output outliers with much greater frequency than other higher-cost sensors such as lidar.

To compensate for the vulnerabilities of using a single ultrasonic sensor, our testbed used an array of multiple sensors. This approach of using an array of sensors provided clear performance enhancement. Multiple objects could be detected separately and simultaneously because the number of independent sensor measurements increased. Furthermore, when detecting a single object, outputs from multiple sensors enable cross-checks that exclude low quality measurements, thereby enhancing the reliability of the distance estimates. As the number of sensors increases, so does the performance of the whole system. Although ultrasonic sensor arrays have been utilized for measuring distances to objects [52,53,54,55,56], they have not been used in outdoor driving situations.

**Figure 1:**
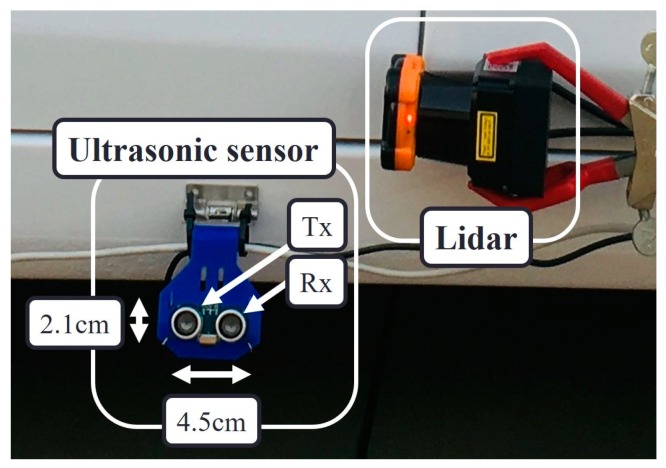
Detailed structure of a single ultrasonic sensor and single-channel light detection and ranging (lidar) in our testbed, attached on the right side of the vehicle.

**Figure 2:**
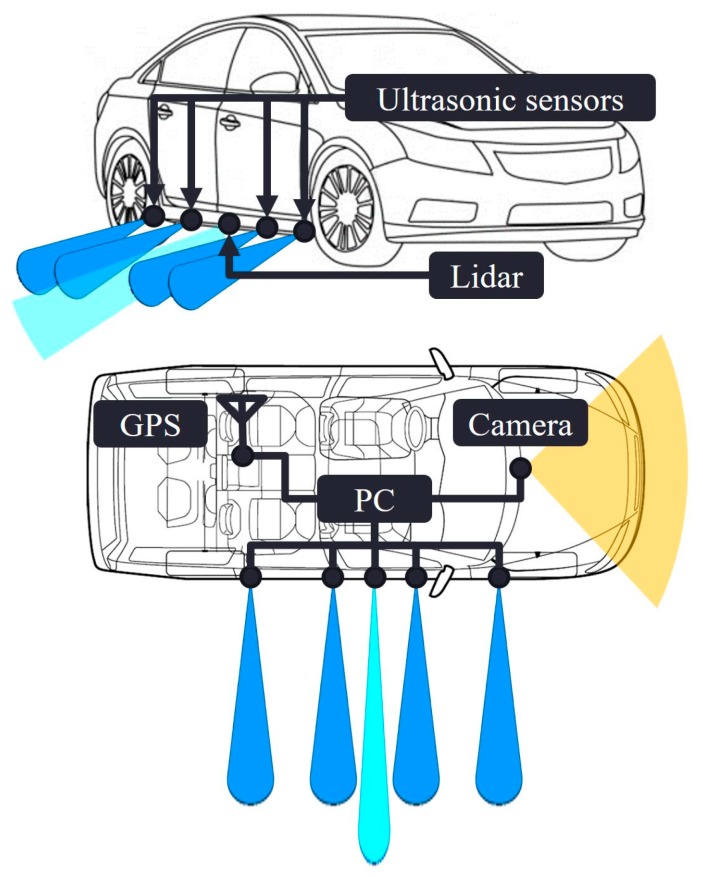
Sensor connection diagram of the testbed and the detection range of each sensor. Lidar, Global Positioning System (GPS), and camera for verification purpose only.

In our curb detection and localization testbed, we equipped the vehicle with four ultrasonic sensors. Three is the minimum number of sensors required to perform the cross-checks. We added one more sensor to evaluate the performance enhancement caused by the fourth ultrasonic sensor. Figure 1 details the types of ultrasonic sensor and single-channel lidar that were attached on the right side of the vehicle. Each ultrasonic sensor was hinge-mounted to enable precise and individual sight line adjustment by changing the tilt angle. The line of sight of every ultrasonic sensor was set parallel to the ground, thus aimed directly at the curb. The entire sensor connection is illustrated in Figure 2.

**Figure 3:**
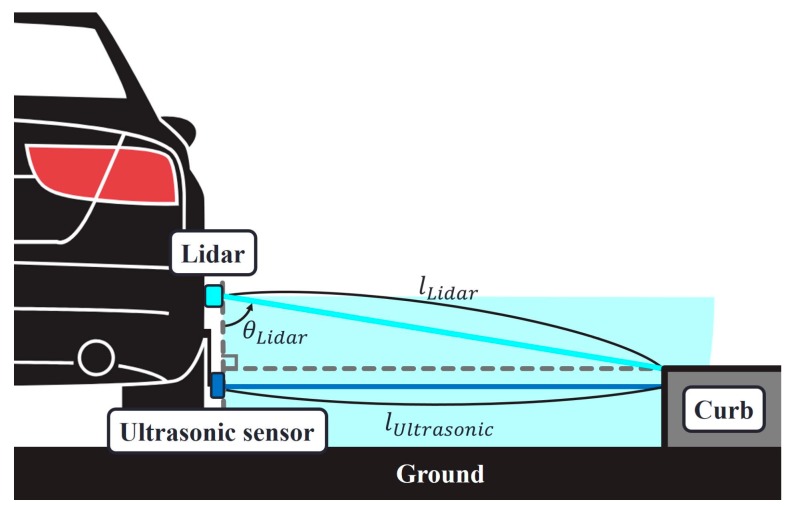
Geometric relationship between the true distance from the ultrasonic sensor to curb (i.e., *l_Ultrasonic_*) and the measured distance and angle using the single-channel lidar (i.e., *l_Lidar_* and *θ_Lidar_*).

The ultrasonic sensors were connected to the USB hub, which was also connected to the vision camera. The USB hub delivered the collected measurement data to a laptop computer. The vision sensor was positioned in front of the vehicle to provide a forward view. It recorded the front view that is used to verify the driving situation. Simultaneously, the GPS receiver collected the GPS position and velocity measurements and recorded the data on the laptop. A single-channel lidar can scan a 2D section in space, and we set the scanning angle from *θ_Lidar_* = 0° to 90°. The *l_Lidar_* and *θ_Lidar_* in Figure 3 were obtained from the raw lidar data and then *l_Ultrasonic_* was calculated, which served as the true distance between the ultrasonic sensor and curb. Again, the lidar, GPS, and vision sensors were not a part of the proposed curb detection and localization system; rather their data were used for performance verification. The operation software that we developed ran the sensors and stored the entire dataset in real time. The collected data were then analyzed by post-processing software, which is discussed in Section 3 and Section 4.

## 3. Distance Estimation Algorithms for Curb Detection and Localization

### 3.1. Simple Averaging and Majority-Voting Algorithms

**Figure 4:**
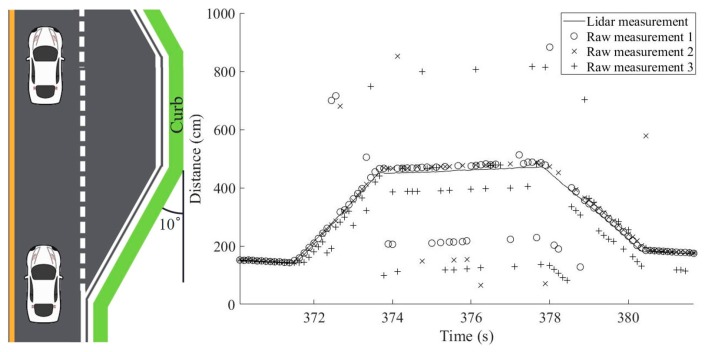
Raw distance measurements from three independent ultrasonic sensors to the curb. Note the frequent outliers: (**left**) driving situation; (**right**) raw measurement data.

The implemented curb-detection testbed provided four distance measurements at each data-acquisition epoch by using the four ultrasonic sensors. The distance-estimation results based on three sensors are presented first and the performance enhancement when all four sensors were used is discussed in Section 5.2. Examples of measurement data from the three sensors are shown in Figure 4 (right). The collected measurements correspond to the situation depicted in Figure 4 (left), where a vehicle drives by a short shoulder of a road. The shoulder has a trapezoidal shape and thus the distance measurements were also expected to form a trapezoidal shape. In the scenario portrayed by Figure 4, the vehicle started to pass the shoulder at 371.5 s and finished at 380.5 s. Raw measurement data points obtained from three sensors are indicated with three different marks (o, x, +). Although distance measurements generally follow the trapezoidal shape of the shoulder, there were a substantial number of outliers that must be properly handled to obtain reliable distance estimates to the curb. 

**Figure 5:**
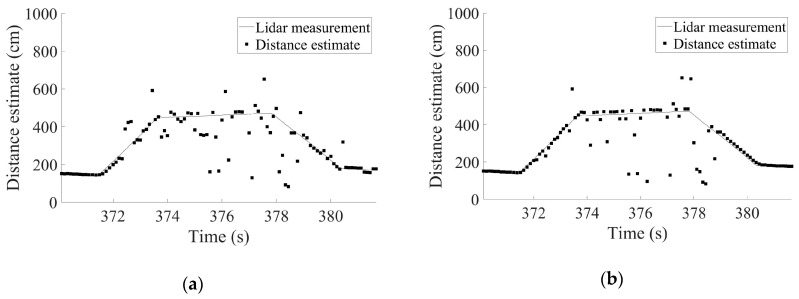
Distance estimates obtained by (**a**) simple averaging algorithm and (**b**) majority-voting algorithm using the raw measurement data from Figure 4.

For a straightforward method to estimate the distance to the curb at each epoch, the three raw distance measurements can be simply averaged. Figure 5a presents the estimated distances calculated by applying the simple averaging algorithm to the raw data from Figure 4. It is almost impossible to recognize the trapezoidal shape of the shoulder in the results of the simple averaging algorithm. The high frequency of measurement outliers in the raw data of Figure 4 produced an averaged output that fluctuated drastically. Figure 5a indicates that the driving situation may not be properly inferred from these erroneous distance estimates.

Because we had three independent measurements at each epoch, a majority-voting algorithm provided a better solution than did the simple averaging algorithm. The closest measurement pair constituted the majority and the remaining single measurement was the minority. Then, the average of the majority measurements determined the estimated distance. The distance estimates derived from the majority-voting algorithm are given in Figure 5b. These results were an improvement on the results of Figure 5a in the sense that the distance estimates followed the trapezoidal shape of the shoulder better; however, there were still many incorrect distance estimates. Further, this simple algorithm does not provide information about which distance estimate is reliable to use for inferring the current driving situation.

### 3.2. Improved Distance Estimation Algorithm Considering Measurement Reliability

To provide reliability information for a distance estimate, we suggested an improved algorithm that classified the measurements of each epoch into three reliability cases: the most reliable case, the less-than-*N*/2-outlier case, and the unreliable case. The distance estimation algorithms for each case are described in this subsection, which extends the simple averaging and the majority-voting algorithms examined in the previous subsection. A general case with *N* ultrasonic sensors attached to a vehicle was assumed. As described in Section 2, *N* = 4, in our testbed but the case of *N* = 3 is first presented. 

#### 3.2.1. Most Reliable Case

When all the distance measurements from *N* sensors at a certain epoch were close enough to each other, those measurements were the most reliable. The standard deviation of *N* measurements in (1) is used as the criterion to determine the closeness.

(1) $$\sigma =\sqrt{\frac{1}{N}\sum_{i=1}^{N}{\left(m_{i}-\mu \right)}^{2}},$$

where *m_i_* is the distance measurement from the *i*-th sensor and *μ* is the average of all *m_i_*. If the standard deviation of measurements is less than the preselected threshold, the case is classified as the most reliable case. Based on the sensor data collected in extensive field tests, the threshold of the standard deviation was set to *σ_reliable_* = 20 cm.

**Figure 6:**
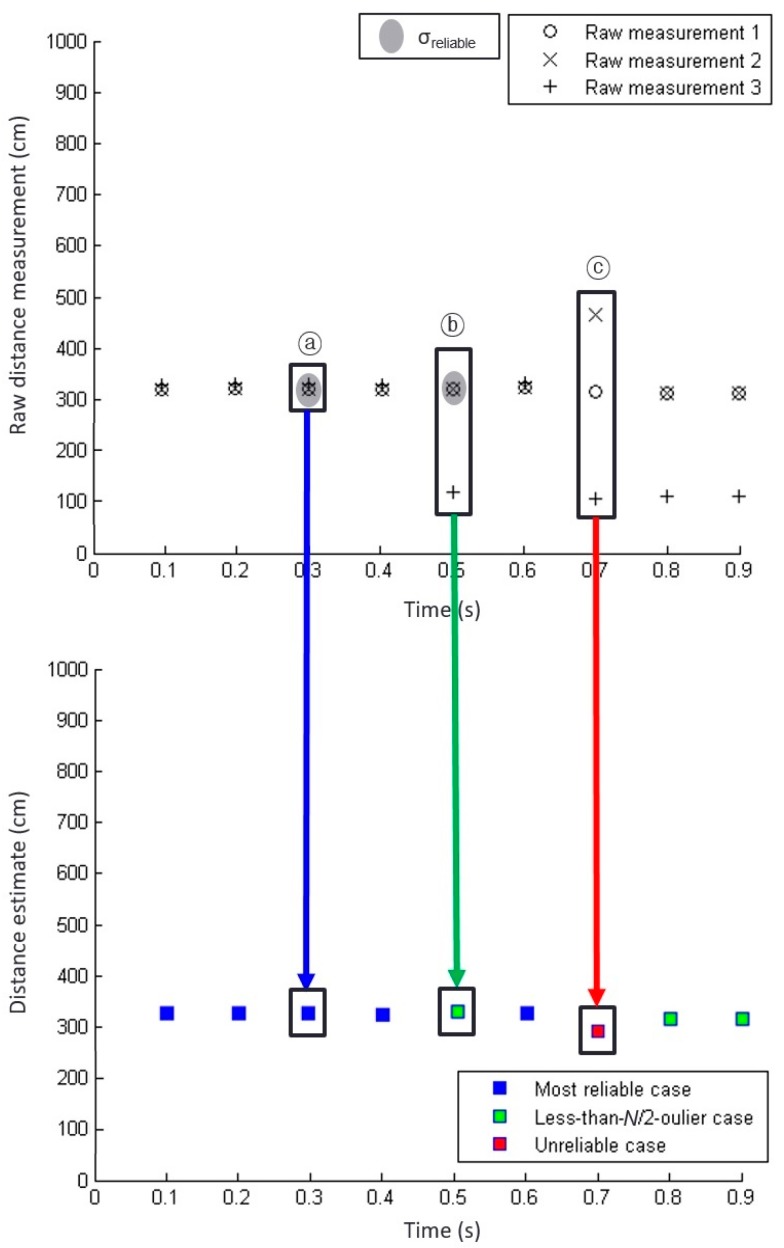
Example of ultrasonic sensor data and distance estimates for three different reliability cases. The (**top**) plot shows raw measurements from three sensors and the (**bottom**) plot shows the distance estimates to the curb based on the raw measurements. Three different estimation methods are used for three different reliability cases as illustrated by three arrows with different colors. The examples at epochs ⓐ, ⓑ, and ⓒ occur in the order of decreasing reliability. The threshold value, *σ_reliable_*, is indicated by grey shades.

This most reliable case corresponds to epoch ⓐ in Figure 6. The raw measurements from three sensors shown in epoch ⓐ of Figure 6 (top) are close to each other. Hence, their standard deviation is less than the threshold, *σ_reliable_*, as indicated by the grey shading in Figure 6 (top). In this case, a simple average of the three raw sensor measurements provides a sufficient estimate of the distance to the curb. The estimated distance at epoch ⓐ is represented with a blue square in Figure 6 (bottom).

#### 3.2.2. Less-than-*N*/2-outlier case

If the measurements of a certain epoch are not classified as the most reliable case, they may belong to the less-than-*N*/2-outlier case, or the single outlier case if *N* = 3. In this case, the majority of the measurements from *N* sensors at a given epoch are reliable even though a few outliers exist.

If *N* = 3, a maximum of three majority vs. minority combinations (i.e., sensors 1 and 2 vs. sensor 3; sensors 2 and 3 vs. sensor 1; sensors 1 and 3 vs. sensor 2) need to be checked. For example, if the measurements of sensors 1 and 2 in the first combination are reliable, as determined by the standard deviation of the measurements, then we do not need to check the second and third combinations. The measurement of sensor 3 was neglected in this case, and our distance estimate is the average of the measurements of sensors 1 and 2. If the majority of the first combination is not reliable, the majority of the next combination is checked until a reliable majority is identified. 

Note that this algorithm differs from the majority-voting algorithm in Section 3.1, where the distance estimate is always provided by averaging the closest measurement pair without the notion of reliability. In the less-than-*N*/2-outlier case, the distance estimate is calculated only if the standard deviation of the measurement pair is less than the threshold of *σ_reliable_*, that is, only if the measurement pair is reliable.

Epoch ⓑ in Figure 6 represents a less-than-*N*/2-outlier case. The measurements at epoch ⓑ do not belong to the most reliable case because their standard deviation was greater than the threshold, *σ_reliable_*, due to an outlier. Excluding the outlier measurement obtained by sensor 3, the standard deviation of the remaining measurements of sensors 1 and 2 was less than *σ_reliable_*. Thus, this case was classified as the less-than-*N*/2-outlier case. The distance estimate at epoch ⓑ averages the measurements from sensors 1 and 2 and is indicated by the green square in Figure 6 (bottom).

If *N* = 5, for example, the maximum number of majority vs. minority combinations to be checked is $\left(\begin{array}{c} 5\\ 4 \end{array}\right)+\left(\begin{array}{c} 5\\ 3 \end{array}\right)=\frac{5!}{4!1!}+\frac{5!}{3!2!}=15$. The distance estimation algorithm for *N* sensors in the less-than-*N*/2-outlier case is given in Algorithm 1. Because the process stops as soon as a reliable majority is found, the algorithm is simple and fast enough for real-time processing.

**Algorithm 1.** Distance estimation algorithm for the less-than-*N*/2-outlier case1 For n from 1 to $\{\begin{array}{l} \lfloor \frac{N}{2}\;(when\;N\;is\;odd)\\ \frac{N}{2}-1\;(when\;N\;is\;even) \end{array}$
2  For all combinations of {*N* − *n* sensors},3   If *σ* {*N* − *n* sensors} < *σ_reliable_*,4    Distance estimate = average of the measurements from {*N* − *n* sensors}.5    Return.6   End.7  End.8 End.

#### 3.2.3. Unreliable Case

Measurements that do not belong to the previous two cases are categorized as the unreliable case. The epoch ⓒ of Figure 6 is an example of the unreliable case. Although we may estimate the distance to the curbs as the average of *N* unreliable measurements, denoted by a red square in Figure 6 (bottom), we do not recommend using this distance estimate for curb detection and localization. Thus, distance estimates derived from measurement sets categorized as unreliable cases were not considered by our implementation.

### 3.3. Outputs from the Improved Distance Estimation Algorithm

**Figure 7:**
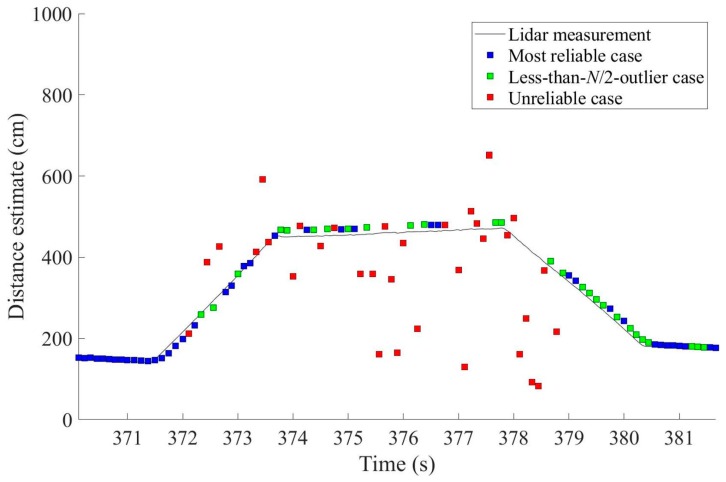
Distance estimation results by the improved algorithm in Section 3.2 with the same raw measurements of Figure 4. This improved algorithm provides a reliability level for each distance estimate among the three reliability cases. However, the availability of reliable distance estimates is only 66.34%.

Our improved distance estimation algorithm was rooted in the simple averaging and the majority-voting algorithms, but it now categorizes the reliability of each distance estimate as one of three reliability levels, enabling users to determine which distance estimates are safe to use for the inference of driving conditions. Figure 7 shows the distance estimation results with corresponding reliability levels based on the application of the proposed algorithm to the same raw measurement data used in Figure 4 and Figure 5. 

Because distance estimates from data categorized as unreliable were discarded, the resulting low availability of reliable distance estimates is a concern for this algorithm. For example, only 66.34% of the epochs in Figure 7 belong to either the most reliable case or the less-than-*N*/2-outlier case. The 66.34% availability of reliable distance estimates may be unacceptably low, impeding the fast and robust decision-making requirements of actual driving situations.

## 4. Proposed Algorithms to Enhance the Availability of Reliable Distance Estimates

**Figure 8:**
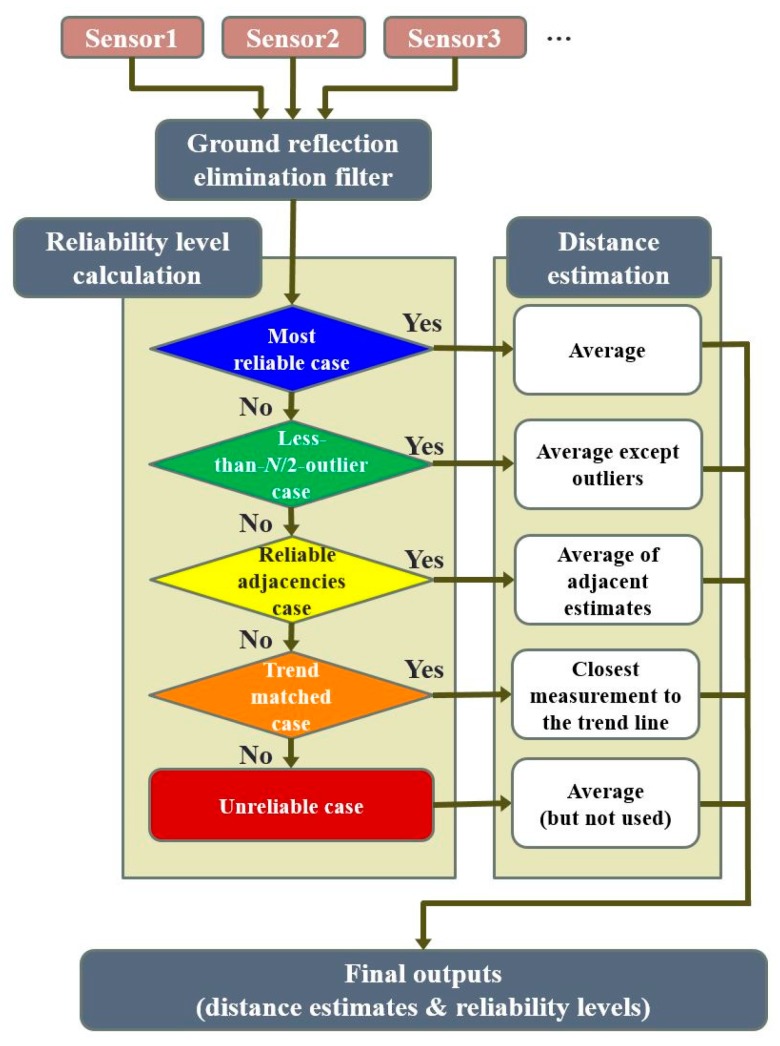
Flow chart of the proposed processing algorithms with *N* ultrasonic sensors for curb detection and localization. In our testbed implementation, *N* = 4 is used.

The improved distance estimation algorithm in Section 3.2 is an extension of the simple averaging and the majority-voting algorithms in Section 3.1. Although this algorithm is straightforward to understand and implement, the low availability of reliable distance estimates is problematic. Therefore, after careful observations of the characteristics of the ultrasonic sensor data in various driving situations, we proposed a series of processing algorithms, outlined in Figure 8, to significantly enhance the availability. Each processing step is described in detail in the following subsections.

### 4.1. Ground Reflection Elimination Filter

**Figure 9:**
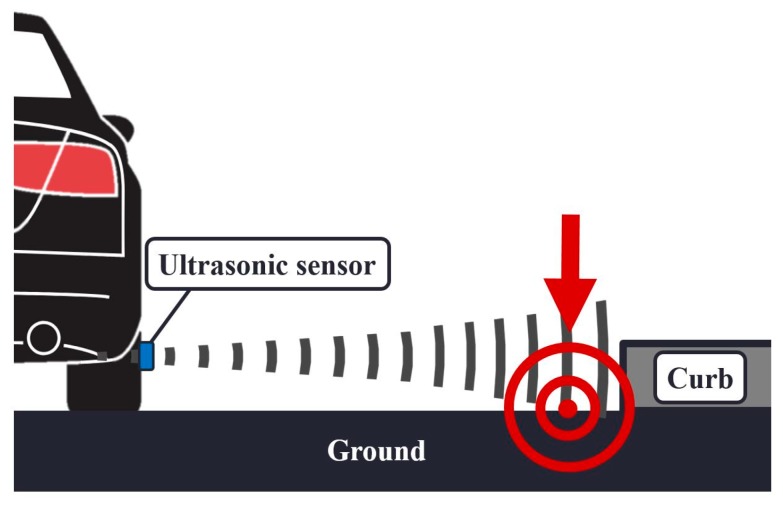
Ground reflection occurs when the distance between the vehicle and the curbs is greater than the distance of the initial setting.

Ground reflection in this paper refers to the undesired reflection of the ultrasonic wave by the ground instead of the curbs. The ultrasonic waves from the sensors were correctly reflected by the curbs at the initial setting because the lines-of-sight of the sensors in our testbed were set to the curb direction. However, during an actual driving situation, the distance between the vehicle and the curbs can differ from that of the initial setting. Then, the ultrasonic wave may touch the ground due to its beam width, as depicted in Figure 9, and the sensors may detect a signal reflected from the ground. This phenomenon causes the sensors to measure the distance to the ground instead of the curbs. Additionally, the vertical fluctuations of a driving vehicle can also cause ground reflection as the height of the sensors relative to the ground changes.

Measurements produced by ground reflection can be distinguished because the measured distance is abnormally smaller than the distance produced by the curbs. Compared to other random measurement outliers, this attribute is unique to ground-reflected measurements. Based on this observation, a ground reflection elimination algorithm for *N* ultrasonic sensors is suggested in Algorithm 2, which is an improved version of our previously presented algorithm [57]. If the majority of the sensors measure distances greater than a selected threshold, and the remaining sensor measurements at the same epoch are less than the threshold, we can determine that the smaller measurements are due to the ground reflection. Because the majority of the sensors provided a better distance estimate to the curb in this situation, the smaller measurements due to ground reflection were replaced by the average distance measurements obtained from the majority of the sensors.

**Algorithm 2.** Ground reflection elimination algorithm1 Separate the *N* sensor measurements of the current epoch into two sets: *A* = {measurements ≥ *d_curb_*}, *B* = {measurements < *d_curb_*}.2 If the size of set *B* is smaller than the size of set A,3  Replace the measurements in *B* with the average value of the measurements in *A*.4 End.

This is a simple, fast, and effective algorithm, but its performance is partially dependent on the selected distance threshold, *d_curb_*. If the threshold is too high, the majority of legitimate distance measurements without ground reflection may not clear the threshold. Then, the algorithm does not eliminate any measurements, even if ground-reflected measurements exist. If the threshold is too small, ground-reflected measurements may be classified as regular measurements and thus will not be eliminated. Given that a reasonable minimum distance to the curbs occurs when the vehicle drives in the middle of the outermost lane, a measurement less than this reasonable minimum distance can be considered as a ground-reflected measurement. In other words, the distance threshold can be determined as follows:

*d_curb_*(*n*) = (*n* − 1)*W_Lane_* + (*W_Lane_* − *W_Vehicle_*)/2 + *W_Gutter_*, (2)

where the vehicle drives in the *n*-th lane from the curb. *W_Lane_* is the width of the lane, *W_Vehicle_* is the width of the vehicle, and *W_Gutter_* is the width of the gutter between the road and curb. In our testbed, *d_curb_*(1) was calculated as 130 cm.

This threshold does not eliminate a ground-reflected measurement if the vehicle is driving closer to the curb than is assumed by the threshold. The remaining ground-reflected measurements were treated as random measurement outliers in the following processing steps. Note that the proposed ground reflection elimination filter does not attempt to eliminate all possible cases of ground reflection, rather, it only eliminates the obvious cases.

### 4.2. Distance Estimation Algorithms with Additional Reliablility Cases

To enhance the availability of reliable distance estimates, we propose two more reliability cases in addition to the three cases described in Section 3.2. The new reliability cases are the reliable adjacencies case and the trend-matched case. These two additional reliability cases effectively survive some measurements that were categorized as the unreliable case in Section 3.2, and consequently increase the availability of reliable distance estimates.

#### 4.2.1. Reliable Adjacencies Case

When the *N* sensor measurements of a certain epoch are not classified as the most reliable case nor the less-than-*N*/2-outlier case, our algorithm postpones its decision on the reliability level of the measurements until the next epoch. At the next epoch, the algorithm checks if the previous epoch belongs to the reliable adjacencies case, which is the third-most reliable case of the five reliability levels depicted in Figure 8.

The reliable adjacencies case occurred when the reliability levels of both adjacent epochs were either the most reliable case or the less-than-*N*/2-outlier case, thus indicating momentary sensor outages between two adjacent epochs with reliable distance estimates. Specifically, if the reliability levels of the measurements at epochs *t* and *t* − 2 were either the most reliable case or the less-than-*N*/2-outlier case, the reliability level of the measurements at epoch *t* − 1 was categorized as the reliable adjacencies case. 

**Figure 10:**
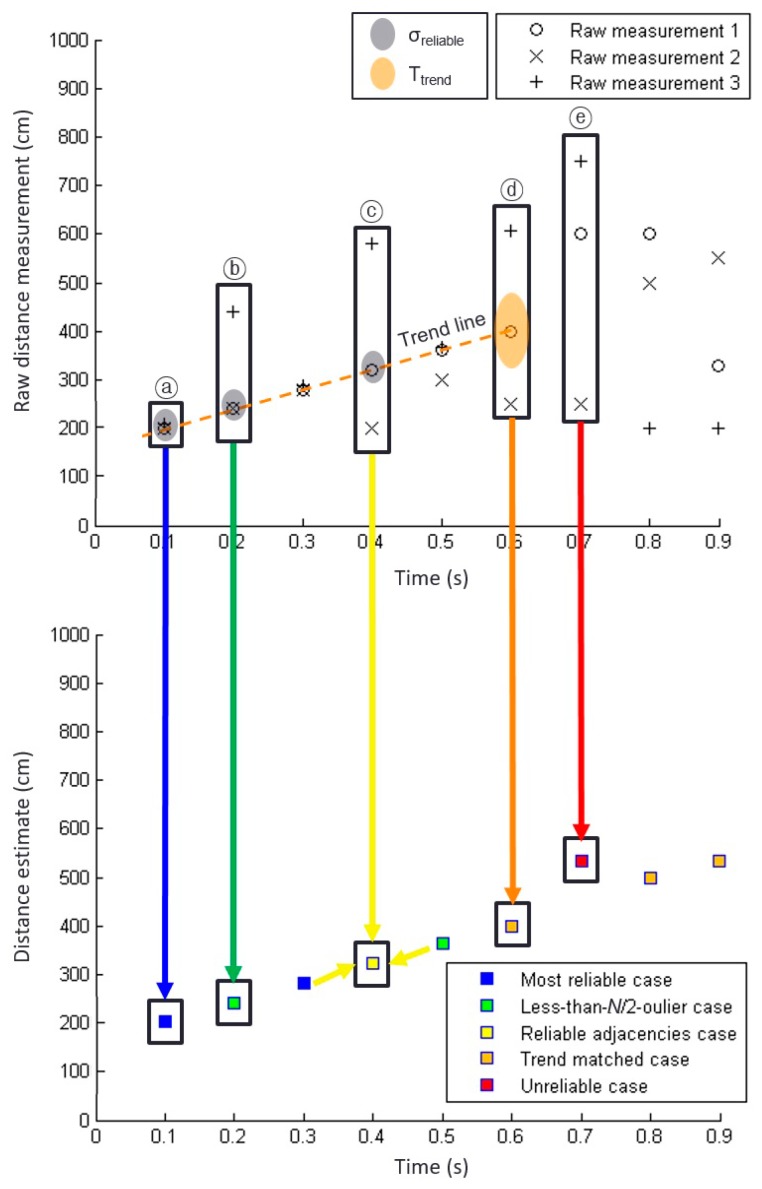
Ultrasonic sensor data and our distance estimates for five different reliability cases. The (**top**) plot shows raw measurements from three sensors and the (**bottom**) plot shows the distance estimates to the curb based on the raw measurements. Five different estimation methods are used for five different reliability cases. The examples at epochs ⓐ, ⓑ, ⓒ, ⓓ, and ⓔ are in the order of decreasing reliability. Two threshold values, *σ_reliable_* and *T_trend_*, are indicated by grey and orange shades, respectively.

The epoch ⓒ of Figure 10 corresponds to the reliable adjacencies case. Because the raw sensor measurements of this epoch were spread, the measurements themselves demonstrated low reliability. However, the measurements of the previous and next epochs belonged to the most reliable case and the less-than-*N*/2-outlier case, which have relatively high reliability. Thus, the unreliable measurements of the epoch ⓒ of Figure 10 were likely due to momentary sensor glitches, and so the average of these measurements will not provide an acceptable distance estimate for the epoch. In the case of reliable adjacencies, our distance estimate is the average of the distance estimates of the preceding and following epochs, that is, the adjacent epochs. The distance estimate marked by a yellow square in Figure 10 (bottom) represents the distance estimation that compensated for the momentary glitches of the epoch ⓒ.

Note that before determining a reliable adjacencies case, there is a fixed delay of one epoch, which is 0.1 s in our testbed with the sensor data rate of 10 Hz. Considering the small lateral movement of a vehicle in 0.1 s under normal circumstances, this delay does not compromise curb detection and localization. Nevertheless, the check for the reliable adjacencies case can be omitted if the 0.1 s delay is critical for a certain application.

#### 4.2.2. Trend-Matched Case

As previously mentioned, other complications in reliable distance estimation result from the noise and frequent outages associated with raw ultrasonic sensor measurements. If we simply ignore all the outages, reliable distance estimations would not be available for the majority of the measurement epochs, rendering the system impractical for curb detection and localization. For example, only 39.60% of the measurement epochs of Figure 4 are categorized as the most reliable case. Thus, reliable distance estimates are not available for 60.40% of time unless other methods are applied. Once the distance estimation method for the less-than-*N*/2-outlier case was applied, we obtained reliable distance estimates for 66.34% of time. 

Our distance estimation method for the reliable adjacencies case in the previous subsection replaced erroneous distance estimates caused by momentary sensor glitches with more reliable estimates based on the measurements of adjacent epochs. If this method was applied in addition to the methods for the most reliable and less-than-*N*/2-outlier cases, the availability of reliable distance estimates from the raw sensor data of Figure 4 increased to 74.26%. This is a drastic improvement upon the 39.60% availability of the base case.

To further improve the availability of reliable distance estimates, we proposed an additional method based on the trend of raw measurement data. As a practical example, the three sensor measurements at the epoch ⓓ of Figure 10 are spread, i.e., this is neither the most reliable nor the less-than-*N*/2-outlier case, and one of the adjacent epochs, epoch ⓔ, shares this status. Thus, the epoch ⓓ of Figure 10 does not belong to the reliable adjacencies case either. 

**Figure 11:**
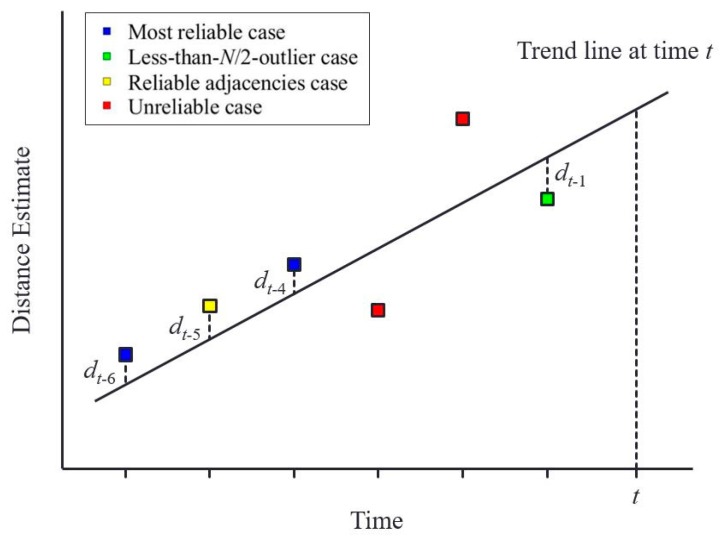
Illustration of constructing a trend line based on the previous reliable distance estimates.

An important observation here is that one sensor measurement, raw measurement 1, of the three measurements at the epoch ⓓ still represents a correct distance measurement while the others experience outages. To capture this correct sensor measurement, we construct a trend line based on the reliable distance estimates belonging to the recent *N_trend_* epochs in the least-squares sense. Figure 11 illustrates a trend line at time *t*; the line is constructed based on the reliable distance estimates of the previous six epochs (i.e., *N_trend_* = 6). The trend line minimizes $d_{t-1}^{2}+d_{t-4}^{2}+d_{t-5}^{2}+d_{t-6}^{2}$ and the unreliable distance estimates at times *t* − 2 and *t* − 3 are not used for calculating the trend line.

The correct sensor measurement is likely to be close to the trend line while the outages are located farther from the trend line, as illustrated by the epoch ⓓ of Figure 10. Therefore, a reasonable distance estimate of this epoch may be based on the single sensor measurement located near to the trend line, rather than on a calculation involving all three spread measurements. 

The distance estimation algorithm with *N* sensors for the trend-matched case is given in Algorithm 3. Six previous epochs (i.e., *N_trend_* = 6) were utilized to construct the trend line, and the threshold *T_trend_* indicating the closeness of each sensor measurement to the trend line was set to 30 cm in our implementation. If multiple measurements were close to the trend line, the algorithm selected the closest one among them. 

**Algorithm 3.** Distance estimation algorithm for the trend-matched case1 Construct a linear line based on the reliable distance estimates belonging to the recent *N_trend_* epochs in the least-squares sense.2 For all the *N* sensor measurements of the given epoch,3  *d* = | each sensor measurement – value of the constructed trend line at the same epoch |.4  If *d* < *T_trend_*,5   *c*[*i*] = *d*.6   *e*[*i*] = corresponding sensor measurement.7   Increase *i* by 1.8  End.9 End.10 *i_smallest_* = index *i* corresponding to the smallest *c*[*i*] value.11 Distance estimate = *e*[*i_smallest_*].

The availability of reliable distance estimates is greatly improved to 92.08% by applying these additional algorithms to the original raw sensor measurements of Figure 4. Recall that the most reliable case after the ground reflection elimination only occurred in 44.55% of the total epochs; this value was 39.60% before the application of the ground reflection elimination filter, and although the distance estimation method for the less-than-*N*/2-outlier case improved the availability of reliable distance estimates to 66.34%, this is still too low for practical application of curb detection and localization. The distance estimation method for the reliable adjacencies case further improved the availability to 74.26%, but the largest improvement was due to the distance estimation method for the trend-matched case with a 17.82 percentage point improvement, which resulted in the 92.08% availability even with the noisy sensor measurements of Figure 4. 

Although our testbed consists of four sensors, that is, *N* = 4, the proposed algorithms are applicable to any number of sensors. Additionally, as more sensors are used, the availability of reliable distance estimates further improves; this is discussed in Section 5.2 by comparing the cases of *N* = 3 and *N* = 4.

### 4.3. Outputs from the Proposed Algorithms

**Figure 12:**
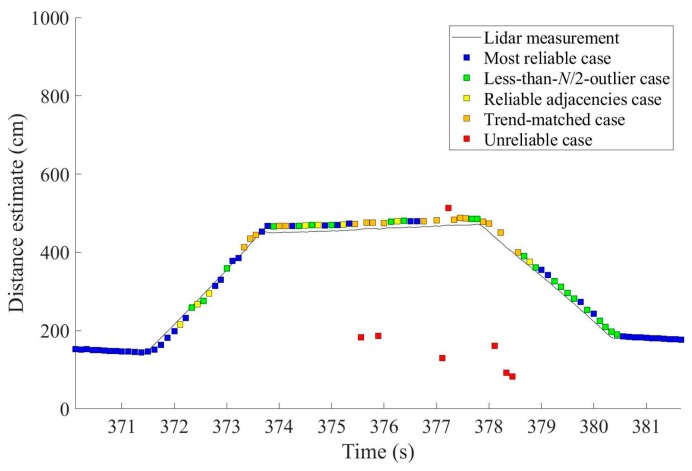
Distance estimates obtained by the proposed algorithms in Section 4 using the same raw sensor measurements of Figure 4. The availability of reliable distance estimates is now 92.08%.

Figure 12 shows an example output of distance estimates after applying all processing algorithms outlined in Figure 8. As throughout the experiment, the raw sensor measurements for Figure 12 are those of Figure 4. Figure 12 demonstrates that the distance estimates clearly follow the shape of the shoulder. Remember that the red squares indicate the unreliable cases and they were not used in curb detection and localization.

## 5. Field Test Results

### 5.1. Field Test Setup

**Figure 13:**
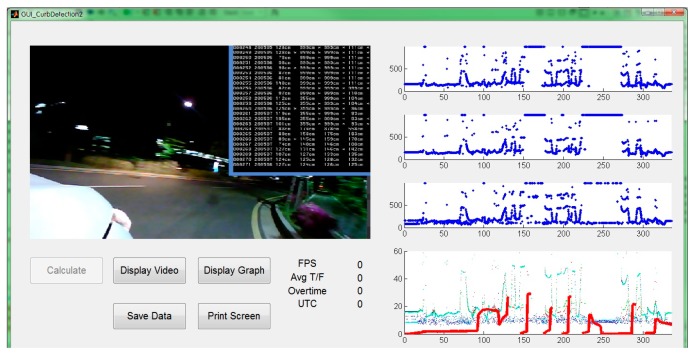
Graphical user interface of our software to collect the raw sensor measurements during field tests.

The hardware setup of the testbed for the field tests was shown in Figure 2. We also developed a software to control the sensors and collect raw measurement data. While the vehicle was in motion, the software stored the raw distance measurements from the ultrasonic sensors and lidar, the position and velocity measurements from the GPS sensor, and the video recording from the vision sensor. The graphical user interface of the developed software is presented in Figure 13.

**Figure 14:**
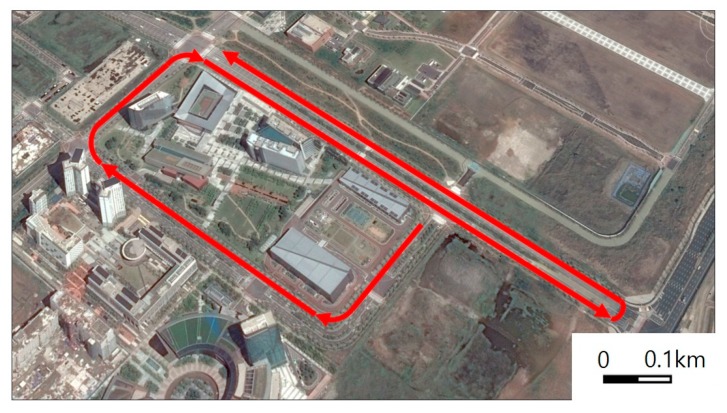
Driving route for the field test in Incheon, Korea.

**Figure 15:**
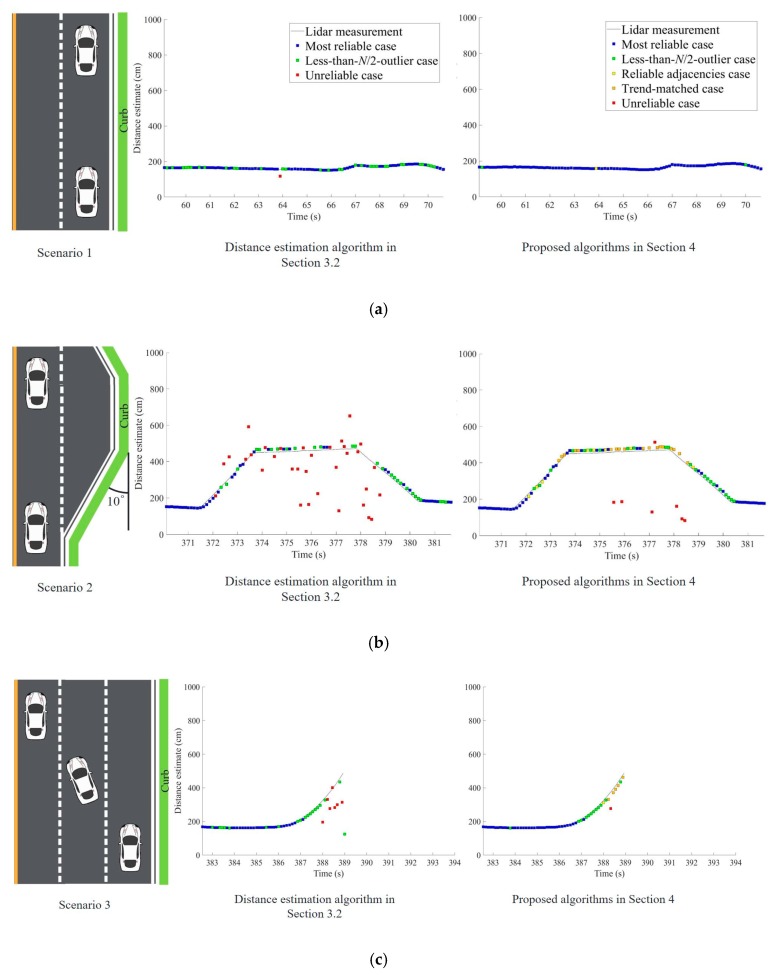

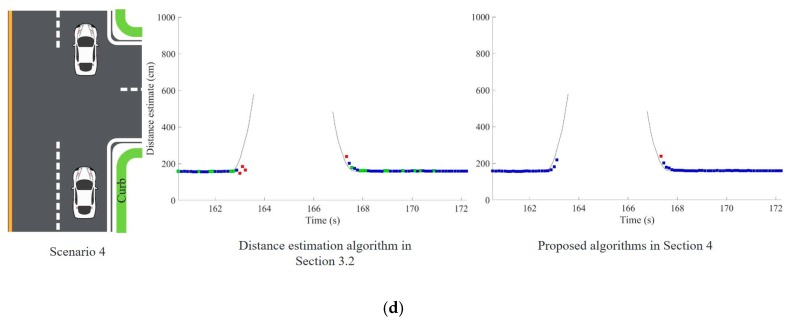
Distance estimation output while (**a**) driving in a straight line beside the curb, (**b**) passing a shoulder on the road, (**c**) changing multiple lanes at once, and (**d**) passing a crossroad.

The driving route in Figure 14 was selected to include representative driving situations and curbs. The route consisted of four driving scenarios. In the first scenario, the vehicle drove in a straight line beside the curb. Thus, the distance estimates to the curb were expected to be almost constant. In the second scenario, the vehicle passed a trapezoidal-shaped shoulder. The distance estimates in this situation were expected to gradually increase and then decrease. Then, the vehicle changed lanes, moving away from the curb in the third driving scenario. In this case, the curb fell out of the range of the ultrasonic sensors. In the fourth scenario, the vehicle passed through a crossroad where the curb suddenly disappeared and reappeared after crossing the intersection. These four driving scenarios are illustrated in Figure 15 next to their respective test results that will be discussed in the next subsection. 

### 5.2. Test Results in Four Representative Driving Situations

Our curb detection and localization system were tested under the four representative driving scenarios. The raw sensor measurements were collected along the driving route depicted in Figure 14. The collected data were post-processed by the algorithms proposed in Section 4 to obtain the results reported in Figure 15. Because the algorithms require low computational power, they presented no problem for real-time implementation. For example, the entire series of algorithms in Figure 8 processed 100 s of raw sensor data using Matlab on a conventional laptop computer in approximately 0.58 s.

The distance estimation algorithm in Section 3.2 is an extension of the majority-voting algorithm. Although this algorithm can provide correct distance estimates if the majority of sensor measurements are correct, the red marks in the middle column of Figure 15 demonstrate that reliable distance estimates are unavailable for many epochs.

The algorithms proposed in Section 4, including the ground reflection elimination filter and the algorithms to handle the reliable adjacencies case and the trend-matched case, solve the problems encountered in Section 3.2. As shown in the right column of Figure 15, unreliable estimates, denoted by red marks, occur at only a few epochs. Even at those epochs, unreliable distance estimates can be discarded because the algorithms have already determined that those estimates were unreliable. Even after neglecting all the unreliable distance estimates, the proposed algorithms provided a very high availability of reliable distance estimates. The benefits of the proposed algorithms were clearly demonstrated, especially in the scenario of passing a shoulder in Figure 15b, where many epochs were classified as the reliable adjacencies or trend-matched cases. Only a few epochs did not have reliable distance estimates to the curbs. 

When the vehicle moves away from the curbs in the different scenarios, the signatures in the distance estimates of Figure 15b–d differ. In Figure 15b, the distance estimates almost linearly increase and remain constant. In Figure 15c, the distance estimates gradually increase and disappear. In Figure 15d, the distance estimates abruptly disappear. Using these unique signatures, the vehicle can now easily classify its surrounding road environment, providing an immediate application of the curb detection and localization system.

**Figure 16:**
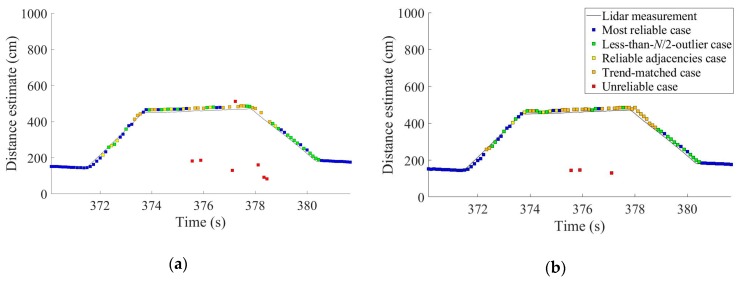
Distance estimates obtained using the proposed algorithms in Section 4 when (**a**) three ultrasonic sensors and (**b**) four ultrasonic sensors were utilized. The availability of reliable distance estimates increased from (**a**) 92.08% to (**b**) 96.04%. The driving scenario is the same as that of Figure 15b.

The field test results up to this point were for the case of utilizing three of the four ultrasonic sensors attached to the vehicle. The proposed algorithms in Section 4 are applicable to any number of ultrasonic sensors, and the improvement of the availability of reliable distance estimates when all the four sensors were used is presented in Figure 16. The availability was improved from 92.05% when *N* = 3 to 96.04% when *N* = 4.

Considering that the distance measurements from the lidar served as the true distances, the curb localization accuracy of the proposed system can be evaluated. The means and standard deviations (SDs) of the distance estimation errors and the root mean square errors (RMSEs) of the simple averaging and majority voting algorithms in Section 3.1 are similar to each other, as shown in Table 1. Although the distance estimates from the majority voting algorithm followed the trapezoidal shape of the shoulder better than those from the simple averaging algorithm in Figure 5, the statistics of the estimation errors are largely influenced by the outliers. Thus, their error statistics are not meaningfully different. Both algorithms provide a 99.01% availability of distance estimates because the algorithms cannot distinguish reliable and unreliable distance estimates, and thus utilize all the estimates. The only case in which these algorithms cannot provide a distance estimate is when all the sensors do not output any distance measurements; this case occurred at only one epoch (Figure 5). 

The proposed algorithms in Section 3.2 and Section 4 demonstrated significantly better accuracies than the simple averaging and majority voting algorithms. The 1-m-level RMSEs of the simple averaging and majority voting algorithms were reduced to a 10 cm level when the proposed algorithms were utilized (Table 1). The proposed algorithms distinguish and discard unreliable distance estimates, and thus the availabilities are relatively lower. Nevertheless, the proposed algorithms in Section 4 provided a 92.08% availability of reliable distance estimates when three ultrasonic sensors were used (i.e., *N* = 3). When one more sensor was added (i.e., *N* = 4), the availability was further improved to a 96.04%.

**Figure 17:**
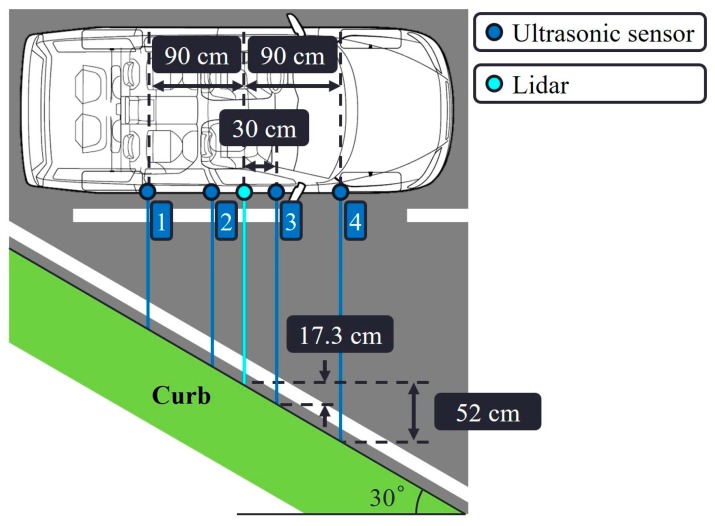
Potential estimation errors due to the orientations of the curb and vehicle.

The proposed system estimated the distance to the curb from the center point of the vehicle where the lidar was attached. If the angle of the curb with respect to the vehicle was large, as illustrated in Figure 17, each ultrasonic sensor measured a different distance. Two sensors measured longer distances while two measured shorter distances than the distance measured at the center point. Recall that the measurements were averaged in the most reliable and less-than-*N*/2-outlier cases (i.e., longer and shorter distances are averaged), and thus the distance estimate at the center point for those cases was not significantly impacted even when the curb angle was large. In addition, as the reliable adjacencies case depended on the most reliable and less-than-*N*/2-outlier cases of adjacent epochs, it was not significantly impacted. However, the distance estimation error of the trend-matched case can grow if the curb angle is large. If the measurement of sensors 3 or 4 in Figure 17 matches with the trend line, an estimation error of 17.3 or 52 cm, respectively, is introduced. During our field test, the curb angle was 10°, which is typical, and the introduced error for the trend-matched case would be 5.3 or 15.9 cm for sensors 3 or 4, respectively. This performance degradation can be reduced if the sensors are placed close to each other. For example, if the separation between ultrasonic sensors is 10 cm, the introduced error for the trend-matched case would be 2.9 or 8.7 cm for sensors 3 or 4, respectively, even when the curb angle is 30°.

## 6. Conclusions

Ultrasonic sensors are widely used low-cost sensors in the automotive industry, and it is tempting to develop a curb detection and localization system using those sensors instead of high-cost distance measuring sensors such as lidar. However, the noisy sensor measurements with very frequent outliers present a challenge to utilizing ultrasonic sensors for this purpose. Although it would be futile to implement such a system using a single ultrasonic sensor, an array of ultrasonic sensors may achieve reliable distance estimations. For example, we can apply typical algorithms such as consistency checking and majority voting if at least three sensors provide raw measurements. This is a good starting point, but it was shown that these methods provided reliable distance estimates to the curbs only 66.34% of the time during our field test passing a shoulder of the road. To further improve the performance of the curb detection and localization system, we suggested a series of processing algorithms that achieved 92.08% availability during the same test scenario with only three ultrasonic sensors. When four ultrasonic sensors were used, the availability was improved to 96.04% with a 13.50 cm RMSE. The performance of our testbed was demonstrated under four representative driving situations and curbs. Because the proposed algorithms are simple, fast, and efficient enough for real-time processing, our proposed method offers a realistic ultrasonic-sensor-based curb detection and localization system, which has not yet been demonstrated in the literature. 

## Figures and Tables

**Table 1:** Comparison of the curb localization accuracy and availability of distance estimates.

	Simple Averaging Algorithm (*N* = 3)	Majority Voting Algorithm (*N* = 3)	Improved Algorithm in Section 3.2 (*N* = 3)	Proposed Algorithms in Section 4 (*N* = 3)	Proposed Algorithms in Section 4 (*N* = 4)
Mean±SD (cm)	−23.62±95.15	−24.66±99.19	6.48±9.89	7.99±10.08	7.89±11.01
RMSE (cm)	97.57	101.73	11.77	12.82	13.50
Availability (%)	99.01	99.01	66.34	92.08	96.04
